# Impact of prophylactic cytomegalovirus immunoglobulin on cytomegalovirus viremia and graft function in ABO-incompatible living donor kidney transplantation: a retrospective analysis

**DOI:** 10.3389/fimmu.2025.1562951

**Published:** 2025-04-28

**Authors:** Linhong Zhong, Shijie Tang, Zhongping Pu, Kai Chen, Wenjia Di, Yifu Hou, Hongji Yang

**Affiliations:** ^1^ Department of Hepatobiliary Surgery, The Affiliated Hospital, Southwest Medical University, Luzhou, Sichuan, China; ^2^ Department of Organ Transplantation, Sichuan Provincial Peoples Hospital, University of Electronic Science and Technology of China, Chengdu, China

**Keywords:** ABO-incompatible kidney transplantation, CMV immunoglobulin, desensitization, graft function, immune deficiency

## Abstract

**Background:**

Cytomegalovirus (CMV) infection poses a significant risk to kidney transplant recipients. CMV immunoglobulin shows promising prophylactic effect, particularly in the context of ABO-incompatible transplants. However, its efficacy in preventing CMV viremia remains underexplored.

**Methods:**

In this retrospective study, we enrolled patients who underwent ABO-incompatible living donor kidney transplantation between May 2021 and September 2023. Prophylactic CMV immunoglobulin was administered at 100 mg/kg weekly for one month in the combined prophylaxis group, while no prophylactic medication was applied in the preemptive therapy group. The primary outcome was measured as the incidence of clinically relevant CMV viremia (CMV DNA **>**10,000 copies/mL) within one year after transplantation. Both groups received standard preemptive therapy with ganciclovir or valganciclovir after diagnosed with clinically relevant CMV viremia.

**Results:**

Prophylactic CMV immunoglobulin significantly reduced clinically relevant viremia incidence compared to preemptive therapy group (16.0% vs. 34.0%, P = 0.04). At the end of the follow-up, the combined prophylaxis group showed higher eGFR (56.40 ± 14.19 vs. 47.30 ± 13.01 mL/min/1.73m², P = 0.0014) and lower serum creatinine (146.5 ± 57.07 vs. 171.2 ± 51.48 µmol/L, P = 0.0274). However, no significant differences in renal function were observed between the groups at1,3, or 6 months post-transplantation.

**Conclusion:**

CMV immunoglobulin represents a promising prophylactic option for reducing clinically relevant CMV viremia incidence and delaying infection onset in ABO-incompatible kidney transplant recipients.

## 1 Introduction

Cytomegalovirus (CMV) infection is a major complication in kidney transplantation, significantly impacting patient survival, prognosis, and graft function (1, 2).With the increasing demand for donor organs among patients with end-stage renal disease, ABO-incompatible (ABOi) kidney transplantation has become widely adopted as a strategy to address donor shortages (3).To successfully perform ABOi transplantation, pretransplant desensitization protocols, which include intensified immunosuppression and antibody removal treatments, are necessary to lower anti-ABO antibody titers and reduce the risk of rejection (4, 5). However, these strategies may increase the risk of CMV infection in ABOi kidney transplant recipients.

Currently, ganciclovir or valganciclovir is widely recommended for the universal prophylaxis and preemptive therapy of CMV infection (5). However, these antiviral medications face significant challenges in the context of prophylaxis, particularly in ABOi transplantation. To begin with, the adverse effects of universal prophylaxis ganciclovir or valganciclovir, particularly the bone marrow suppression and nephrotoxicity, often lead to treatment discontinuation in ABOi transplant recipients (6, 7). Secondly, prolonged use of antiviral drugs may induce resistance, increasing the risk of CMV recurrence and indirectly threatening graft survival (8). Moreover, High economic costs, poor adherence to therapy, and limited drug availability further exacerbate the difficulties of CMV management in ABOi kidney transplant recipients (7, 9). In light of these challenges, the potential role of CMV immunoglobulin (CMVig) as a prophylactic agent in ABOi kidney transplantation remains underexplored, with limited data available to support its efficacy and application in this specific setting.

Notably, before the advent of antiviral drugs, CMVig had already been used to some extent for preventing CMV infection in kidney transplant recipients (10, 11). Recent studies and supplementary preclinical data have provided supportive evidence, reigniting interest in the potential of CMVig among ABOi transplant populations (12, 13). Given the unique immunological status and therapeutic needs of ABOi kidney transplant recipients, more personalized and intensive prophylactic measures are warranted (14, 15). If short-term CMVig administration can significantly reduce CMV infection rates or delay infection events, this could effectively alleviate the burden of ganciclovir/valganciclovir treatment. This would further address long-standing concerns of immunosuppressive load and drug-related adverse effects in ABOi kidney transplant recipients.

While CMVig demonstrates promising potential, its precise application protocols and long-term efficacy in ABOi kidney transplantation remain to be fully elucidated. This study aims to explore the role of CMVig in preventing CMV infections post-ABOi kidney transplantation, evaluate its effectiveness and safety under unique immune conditions, and provide a theoretical and clinical basis for optimizing infection management strategies in ABOi kidney transplantation.

## 2 Materials and methods

### 2.1 Patients and study design

**Figure 1:**
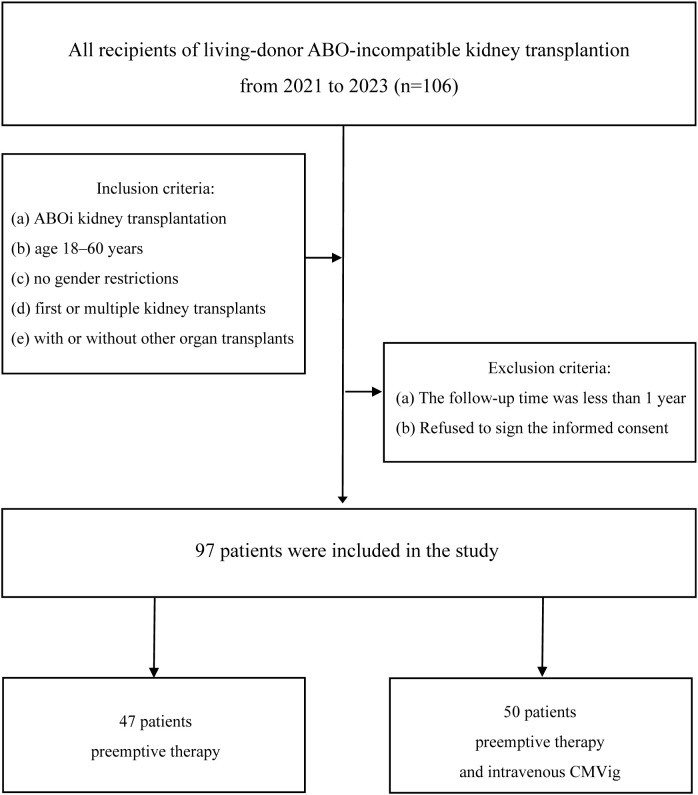
Study cohort and workflow. Patients were divided into two groups: preemptive therapy group (n=47) and combined prophylaxis group (preemptive therapy+CMVig, n=50). ABOi, ABO-incompatible; CMVig, Cytomegalovirus immunoglobulin.

This retrospective, single-center study analyzed patients who underwent ABOi kidney transplantation at our hospital between May 2021 and September 2023. The study design and workflow are illustrated in **Figure 1**. Inclusion criteria included: (a) ABOi kidney transplantation, (b) age 18–60 years, (c) no gender restrictions, (d) first or multiple kidney transplants, and (e) with or without other organ transplants. Patients with follow-up periods of less than one year or those who declined informed consent were excluded. The study adhered to the Declaration of Helsinki and was approved by the hospital’s Ethics Committee and was approved by the Ethics Committee of the Sichuan Provincial Peoples Hospital (No. 20244631).

Patients were divided into two groups based on their postoperative CMV infection prevention protocol through patient-physician shared decision-making. The preemptive therapy group(PET group) received standard preemptive therapy with ganciclovir or valganciclovir initiated upon the detection of clinically relevant CMV viremia (CMV DNA >10,000 copies/mL). The combined prophylaxis group (CMVig group), defined as patients receiving CMV immunoglobulin (CMVig) in addition to preemptive therapy, was administered an additional prophylactic regimen of CMV immunoglobulin (CMVig) administered at a dose of 100 mg/kg weekly for one month, starting on the first postoperative day. Outcomes assessed during a one-year follow-up included the incidence of clinically relevant CMV viremia, renal function (eGFR and serum creatinine), and postoperative complications (**Figure 1**).

### 2.2 ABO desensitization protocol and immunosuppressive regimen

**Figure 2:**
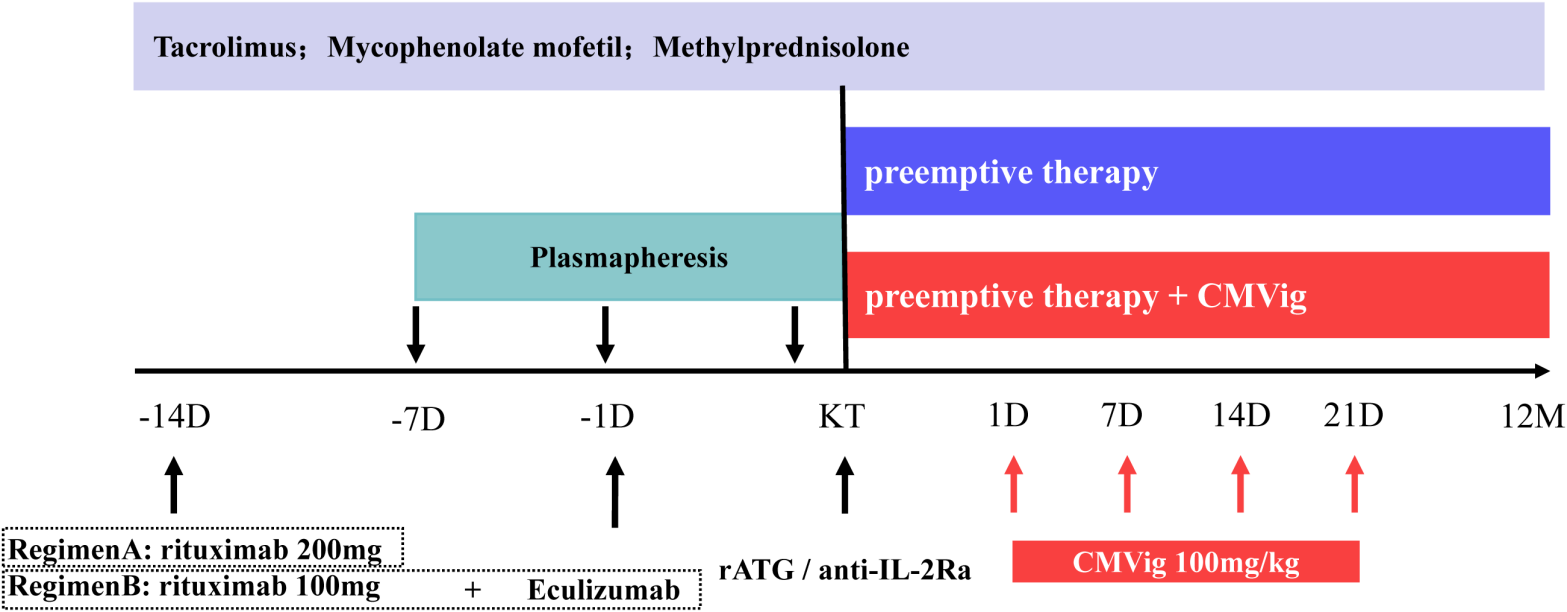
ABO desensitization protocols and immunosuppressive regimen. CMVig, Cytomegalovirus immunoglobulin; KT, kidney transplantation; rATG, rabbit anti-thymocyte globulin; anti-IL-2Ra, basiliximab; D, day; M, month.

Desensitization was achieved using plasmapheresis and monoclonal antibody therapy. Plasmapheresis was initiated seven days before transplantation (Day -7) with the goal of reducing anti-ABO antibody titers (IgM and IgG) to ≤1:16 prior to surgery. Monoclonal antibody therapy is carried out in two regimens. the first regimen consists of a single 200 mg dose of CD20 monoclonal antibody (administered two weeks preoperatively). The second regimen combines a reduced 100 mg dose of CD20 monoclonal antibody (administered two weeks preoperatively), with a complement C5 inhibitor, eculizumab (600 mg for patients <60 kg and 900 mg for patients ≥60 kg, administered one day before surgery) (**Figure 2**). Two weeks prior to transplantation, patients began a triple immunosuppressive regimen consisting of a calcineurin inhibitor, an antimetabolite (mycophenolate mofetil), and oral prednisone. Induction therapy involved rabbit anti-thymocyte globulin or basiliximab.

### 2.3 Surgical procedure

Living donor organ procurement followed strict legal and ethical protocols, including approval from the hospital Ethics Committee and provincial health authorities. Donors were categorized as genetically related (e.g., parents, siblings, nephews/nieces) or non-genetically related (spouses). The methods for living donor nephrectomy are primarily classified into two types: open donor nephrectomy and laparoscopic donor nephrectomy via the retroperitoneal approach (**Supplementary Table S1**).

### 2.4 CMV infection monitoring and prevention

Clinically relevant CMV viremia was defined as CMV DNA levels exceeding 10,000 copies/mL. CMV DNA quantification was performed using quantitative nucleic acid testing with the Human Cytomegalovirus Nucleic Acid Quantitative Detection Kit (PCR-fluorescence probing; DAAN Gene Co., Ltd., China). Whole blood samples were analyzed. Postoperative CMV DNA levels were monitored weekly for the first three months, then monthly until one year. Prevention strategies included either standard preemptive therapy alone or combined with prophylactic CMVig. In the PET group, antiviral treatment was initiated upon detecting a viral load exceeding the threshold(CMV DNA >10,000 copies/mL). Patients received either valganciclovir (900 mg, twice daily, with dosage adjusted based on renal function) or ganciclovir (5 mg/kg, every 12 hours, with dosage adjusted based on renal function). The treatment duration was at least two weeks and continued until viral replication was completely eradicated. To evaluate response to preemptive therapy, weekly quantitative CMV DNA monitoring was performed. In the CMVig group, in addition to the preemptive therapy protocol, prophylactic CMVig (Human Immunoglobulin for Intravenous Injection, Shandong Taibang Biological Products Co., Ltd., China; potency: 721 IU/mL) was administered at 100 mg/kg (1442 IU/kg) weekly for one month, starting on the first postoperative day.

### 2.5 B cells monitoring and other outcomes

Peripheral blood B-cell counts were analyzed by flow cytometry (Beckman Coulter CytoFLEX) using whole blood staining with PE-Cy7-conjugated anti-CD19 (clone J3-119; Beckman Coulter).Results were expressed as absolute cell counts (cells/μL) and relative percentages of total lymphocytes. Data were retrospectively collected from the following time points: 14 days pre-transplant(day of rituximab administration), day of transplantation, and postoperative follow-ups. We also followed the rejection and infection-related events. Rejection (TCMR or ABMR) was diagnosed via Banff 2019 criteria. Infections were defined as: pulmonary infections (clinical symptoms and radiological/microbiological confirmation), BK virus infections (viruria/viremia via PCR, viruria ≥1×10_7_ copies/mL, viremia ≥1×10_4_ copies/mL), and herpesvirus infections (PCR/serology).

### 2.6 Statistical analysis

All statistical analyses were performed using SPSS version 26.0 and R version 4.0. Continuous variables are presented as mean ± standard deviation (SD) or median (interquartile range, IQR), and were compared using either t-tests or Mann-Whitney U tests, as appropriate. Categorical variables were analyzed using chi-square or Fisher’s exact tests. Kaplan-Meier survival analysis with log-rank tests was used to compare the incidence of clinically relevant CMV viremia between the groups. To identify factors influencing CMV viremia, both univariate and multivariate logistic regression analyses were conducted. Univariate analysis was first performed to assess the relationship between each individual factor and CMV viremia, including the following variables: group, gender, induction therapy type, body mass index, dialysis duration, pretransplant anti-A/B antibody titer, renal glomerular filtration rate, warm ischemia time, cold ischemia time, and HLA mismatch. Subsequently, multivariate analysis was used to identify independent factors significantly associated with CMV viremia, adjusting for potential confounders. Given its potential clinical significance, induction therapy type was forced into the multivariate regression model regardless of its univariate statistical results. A backward stepwise regression approach was employed to select the most relevant variables for the multivariate model. Changes in renal function over time were assessed using repeated measures ANOVA. A two-tailed P-value of < 0.05 was considered statistically significant.

## 3 Results

### 3.1 Patient characteristics

A total of 106 patients underwent ABOi kidney transplantation, with 50 patients in the CMVig group and 47 in the PET group. Nine patients were excluded due to insufficient follow-up (n = 4) or refusal to participate (n = 5). Baseline characteristics, as summarized in **Table 1**, were comparable between the two groups. The mean recipient age was 37.8 ± 9.0 years in the CMVig group and 35.5 ± 10.0 years in the PET group (P = 0.234). The median dialysis duration was 12.0 months (IQR: 3.0–24.0) in the CMVig group and 8.0 months (IQR: 5.0–12.0) in the PET group (P = 0.594). The mean donor kidney glomerular filtration rate (GFR) was 41.7 ± 5.4 mL/min in the CMVig group and 42.9 ± 5.6 mL/min in the PET group (P = 0.280). Both groups had a median of two HLA mismatches (IQR: 2–3 in the CMVig group vs. 1–3 in the PET group, P = 0.100). Pre-transplant anti-A/B antibody titers were similar, with a median titer of 1:4 (IQR: 1:4–1:8) in both groups (P = 0.836). All donor-recipient pairs were CMV seropositive (D+/R+). Additionally, there were no significant differences in other parameters, including gender distribution, warm ischemia time, or cold ischemia time (all P > 0.05).

**Table 1:** Patient Characteristics.

	preemptive therapy(n=47)	preemptive therapy+CMVig(n=50)	p-value
Recipient’s characteristics			
Age (years, mean ± SD)	37.8 (± 9.0)	35.5 (± 10.0)	P=0.234
Male/female (n)	30/17	31/19	P=0.852
BMI (kg/m², median, IQR)	22.1 (19.8-24.2)	21.2 (18.7-23.4)	P=0.175
Time on dialysis (months, median, IQR)	8.0 (5.0-12.0)	12.0 (3.0-24.0)	P=0.594
Comorbidity (n)			
hypertension	28 (59.6%)	30 (60.0%)	P=0.966
diabetes	7 (14.9%)	15 (30.0%)	P=0.076
cardiovascular disease	4 (8.5%)	5 (10.0%)	P=0.801
hepatitis B	6 (12.8%)	8 (16.0%)	P=0.651
Diagnosis of end stage renal disease (n)			P=0.327
diabetic nephropathy	6 (12.8%)	13 (26.0%)	
hypertensive nephropathy	22 (20.4%)	20 (40.0%)	
glomerulonephritis	8 (17.0%)	4 (8.0%)	
others and undetermined	11 (23.4%)	13 (26.0%)	
Blood group (n)			P=0.549
A→B	7 (14.9%)	6 (12.0%)	
A→O	9 (19.1%)	9 (18.0%)	
B→A	12 (25.5%)	11 (22.0%)	
B→O	7 (14.9%)	11 (22.0%)	
AB→A	9 (19.1%)	8 (16.0%)	
AB→B	3 (6.4%)	5 (10.0%)	
Donor specific antibodies (n)	2	1	
Donor characteristics			
Age (years, mean ± SD)	53.7 (± 7.1)	52.0 (± 9.1)	P=0.304
Male/female (n)	21/26	25/25	P=0.600
Renal GFR (ml/min, mean ± SD)	42.9 (± 5.6)	41.7 (± 5.4)	P=0.280
Desensitization Protocol and Immunosuppressive regimen (n)			
Desensitization regimen A/B	47/0	46/4	P=0.118
Basiliximab/anti-thymocyte globulin	7/40	3/47	P=0.320
Surgery-related statistics			
Donor/recipient serostatus (D/R)			
D+/R+ (n)	47 (100%)	50 (100%)	
Peak baseline anti-A/B antibody titer (median, IQR)	1:32 (1:16-1:128)	1:32 (1:16-1:80)	P=0.698
Pretransplant anti-A/B antibody titer (median, IQR)	1:4 (1:4-1:8)	1:4 (1:4-1:8)	P=0.836
HLA mismatch (n, median, IQR)	2 (1-3)	2 (2-3)	P=0.100
Warm ischemia time (min, median, IQR)	3 (2-3)	2 (2-3)	P=0.114
Cold ischemia time (min, median, IQR)	168.0 (± 50.4)	157.4 (± 44.1)	P=0.269

CMVig = Cytomegalovirus immunoglobulin, SD = Standard deviation, BMI = Body mass index, IQR = Interquartile range, GFR = Glomerular filtration rate, HLA = human leukocyte antigen.

### 3.2 CMV infection outcomes

**Figure 3:**
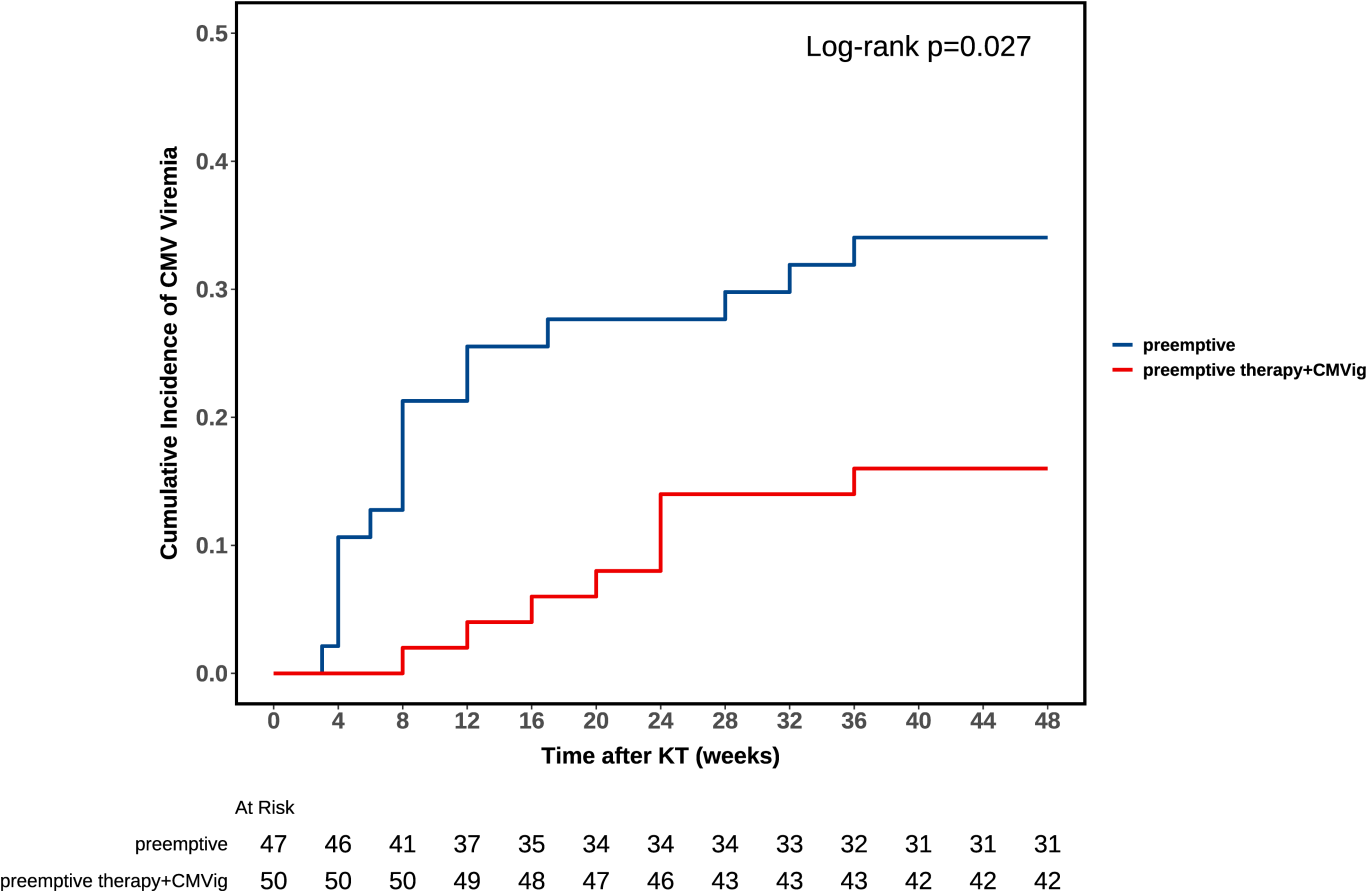
Kaplan-Meier analysis of CMV viremia-free survival. The curve compares clinically relevant CMV viremia-free survival rates between the combined group (preemptive therapy + CMVig) and the preemptive therapy group. A significant difference was observed (log-rank test, P = 0.027).

During the one-year follow-up, the clinically relevant CMV viremia occurred in 24.7% (24/97) of patients. The incidence was significantly lower in the CMVig group (16.0%, 8/50) compared to the PET group (34.0%, 16/47; P = 0.04). Notably, no cases of CMV end-organ disease were observed in either group. Furthermore, the median time to post-transplant infection was 8 weeks (IQR: 4-15.75) in the PET group versus 22 weeks (IQR: 13-24) in the CMVig group (P = 0.032). Kaplan-Meier analysis demonstrated clear divergence in cumulative CMV progression between groups (**Figure 3**). The log-rank test confirmed statistical significance (P = 0.027) (**Figure 3**). Regarding treatment duration, the median course of preemptive antiviral therapy was shorter in the CMVig group (2 weeks, IQR: 2-4) compared to the PET group (3.5 weeks, IQR: 2-4), although this difference did not reach statistical significance (P = 0.383).

To explore factors associated with CMV viremia, univariate logistic regression analysis was conducted. The results indicated that patients in the CMVig group (i.e., CMVig intervention) had a significantly lower risk of clinically relevant CMV viremia compared to the PET group (OR = 0.37, 95% CI: 0.13–0.95, P = 0.043). Univariate logistic regression analysis also identified three independent risk factors: higher pre-transplant anti-A/B antibody titers (OR = 1.13, 95% CI: 1.02–1.25, P = 0.014), prolonged warm ischemia time (OR = 2.06, 95% CI: 1.18–3.69, P = 0.012), and a greater number of HLA mismatches (OR = 2.25, 95% CI: 1.45–3.77, P < 0.001). Different induction therapy, Gender, Body mass index, dialysis duration, renal GFR, and cold ischemia time did not show significant associations with CMV viremia (all P > 0.05) (**Table 2**). Multivariate logistic regression analysis further confirmed the independent impact of these factors. CMVig intervention was a strong protective factor, significantly reducing the risk of CMV viremia (adjusted OR = 0.19, 95% CI: 0.04–0.68, P = 0.016). Conversely, higher pre-transplant anti-A/B antibody titers (adjusted OR = 1.19 per unit increase, 95% CI: 1.04–1.38, P = 0.016), prolonged warm ischemia time (adjusted OR = 2.67, 95% CI: 1.24–6.46, P = 0.018), and increased HLA mismatches (adjusted OR = 2.78, 95% CI: 1.63–5.47, P < 0.001) were identified as independent risk factors for CMV viremia (**Table 2**).

**Table 2:** Univariate and Multivariate Analyses of Factors Influencing cytomegalovirus viremia.

Characteristic	Univariable			Multivariable		
	OR	95% CI	p-value	OR	95% CI	p-value
Group						
preemptive therapy	Referent	—		—	—	
preemptive therapy+CMVig	0.37	0.13, 0.95	0.043	0.19	0.04, 0.68	0.016
Gender						
male	Referent	—		—	—	
female	0.36	0.11, 1.00	0.063	0.28	0.06, 1.05	0.075
Induction therapy						
basiliximab	Referent	—		—	—	
rabbit anti-thymocyte globulin	0.74	0.19, 3.68	0.685	0.95	0.12, 10.76	0.962
Body mass index (kg/m²)	1.06	0.94, 1.21	0.335			
Time on dialysis (months)	0.99	0.95, 1.03	0.645			
Pretransplant anti-A/B antibody titer	1.13	1.02, 1.25	0.014	1.19	1.04, 1.38	0.016
Renal glomerular filtration rate	1.04	0.95, 1.13	0.386			
Warm ischemia time (min)	2.06	1.18, 3.69	0.012	2.67	1.24, 6.46	0.018
Cold ischemia time (min)	1.00	0.99, 1.01	0.683			
HLA mismatch (n)	2.25	1.45, 3.77	<0.001	2.79	1.63, 5.47	<0.001

OR, Odds ratio; CI, Confidence interval; CMVig, Cytomegalovirus immunoglobulin; HLA, human leukocyte antigen.

### 3.3 Renal allograft function

**Figure 4:**
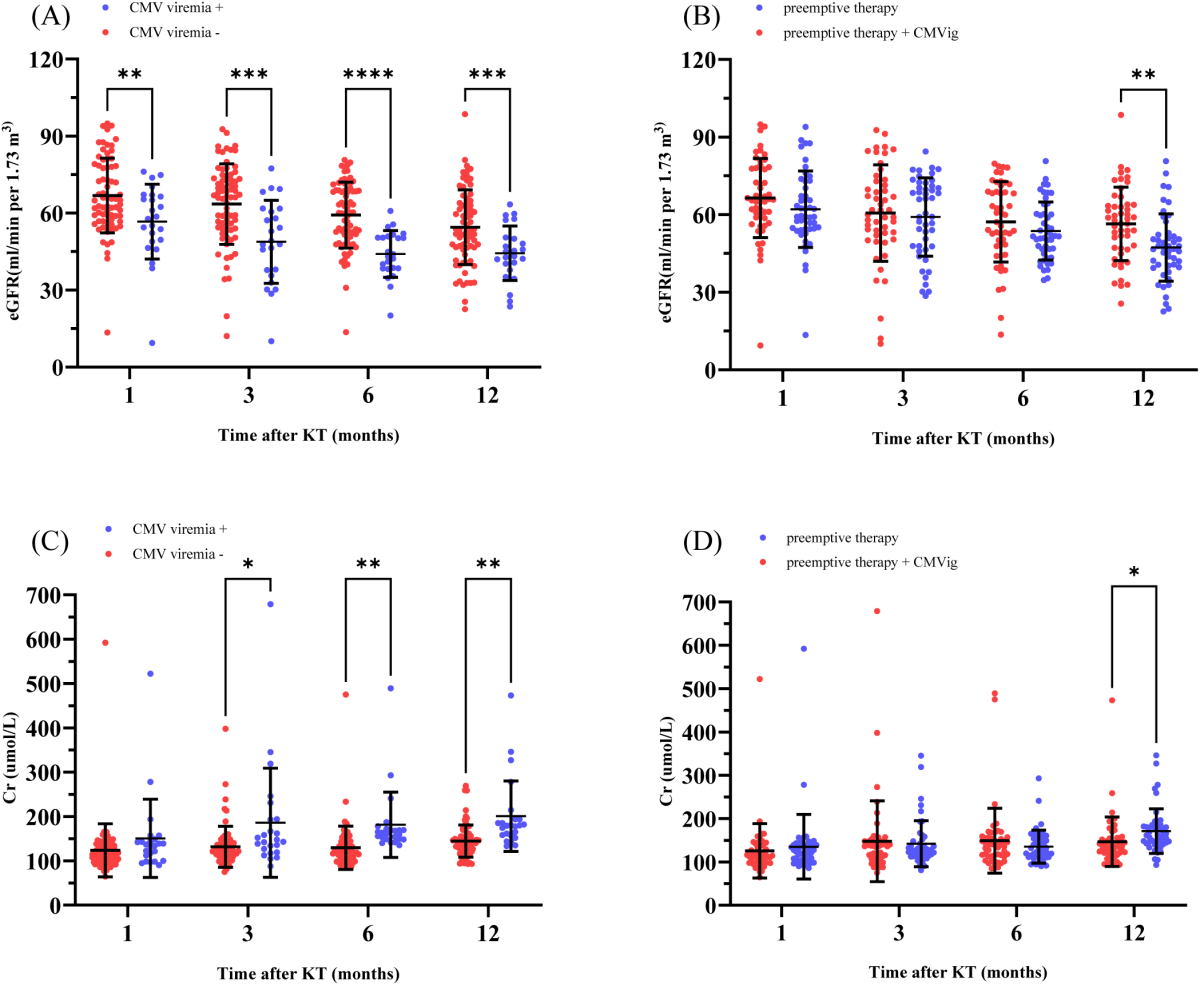
Renal allograft function over time. **(A)** eGFR comparison between patients with and without clinically relevant CMV viremia post-transplant. **(B)** eGFR comparison between the two groups. **(C)** Cr levels in patients with and without clinically relevant CMV viremia. **(D)** Cr levels in the two groups. eGFR, estimated Glomerular Filtration Rate; Cr, Serum creatinine; CMVig, Cytomegalovirus immunoglobulin; KT, kidney transplantation. *P<0.05; **P<0.01; ***P<0.001; ****P<0.0001.

CMV infection impacts kidney function in transplant patients. In this study, renal allograft function was consistently poorer in clinically relevant CMV viremia-positive patients compared to viremia-negative patients at multiple time points during the follow-up period. Specifically, the mean estimated glomerular filtration rate (eGFR) in CMV viremia-positive patients was significantly lower than in viremia-negative patients at 1, 3, 6, and 12 months post-transplant: 56.68 ± 14.59 vs. 66.84 ± 14.54ml/min/1.73m² (P = 0.0051) at 1 month, 48.82 ± 16.16 vs. 63.49 ± 15.70 ml/min/1.73m² (P = 0.0004) at 3 months, 44.06 ± 9.09 vs. 59.23 ± 12.85 ml/min/1.73m² (P < 0.0001) at 6 months, and 44.36 ± 10.59 vs. 54.50 ± 14.54ml/min/1.73m² (P = 0.0005) at 12 months. Similarly, serum creatinine levels also differed significantly between the two groups, being higher in clinically relevant CMV viremia-positive patients at 3, 6, and 12 months: 185.95 ± 122.98 vs. 131.58 ± 46.26 µmol/L (P = 0.0443) at 3 months, 181.38 ± 73.79 vs. 129.54 ± 48.94 µmol/L (P = 0.0031) at 6 months, and 200.76 ± 79.45 vs. 144.60 ± 36.15 µmol/L (P = 0.0025) at 12 months (**Figures 4A, C**). A comparison of renal allograft function between the two groups over the one-year follow-up period demonstrated significant differences (**Figures 4B, D**). At 12 months post-transplant, the eGFR in CMVig group was significantly higher than in the PET group (56.40 ± 14.19 vs. 47.30 ± 13.01 ml/min/1.73m², P = 0.0014). Additionally, serum creatinine levels in the CMVig group were significantly lower than those in PET group at the same time point (146.52 ± 57.07 vs. 171.22 ± 51.48 µmol/L, P = 0.0274) (**Figure 4**). Although urinary protein positivity rates increased over time in both groups, the PET group consistently exhibited higher positivity rates at 1,3,6 and 12 months post-transplant. However, these differences were not statistically significant (**Supplementary Figure S1**).

### 3.4 B cells reconstitution

**Figure 5:**
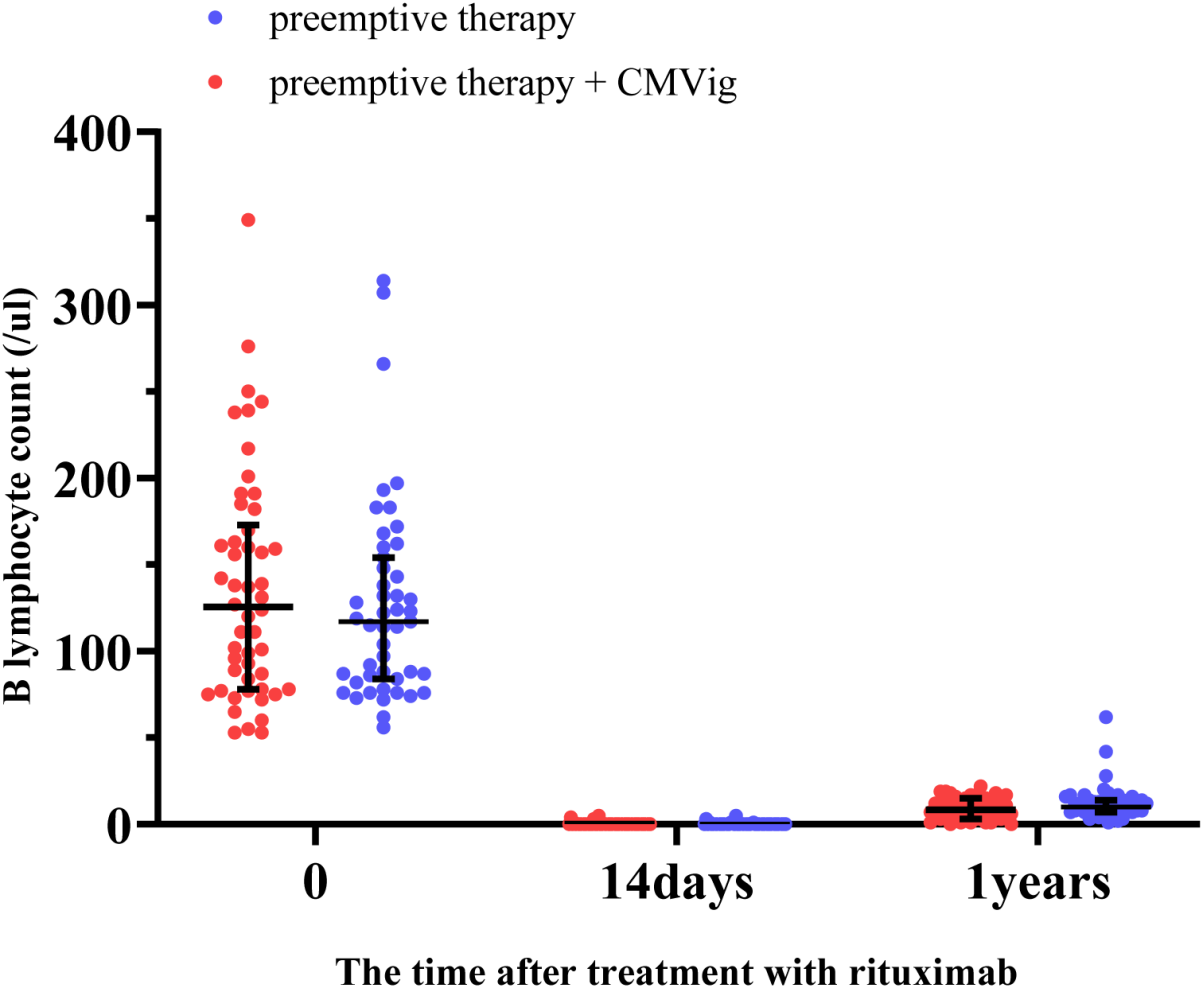
B lymphocyte depletion and reconstitution after rituximab treatment. This figure demonstrates the changes in peripheral blood B lymphocyte counts (cells/μL) in the two groups over time.

The dynamics of B cells recovery following rituximab treatment were assessed. Before treatment, the median B cell count was 117 cells/μL (IQR: 84–154) in the PET group and 125 cells/μL(IQR: 78–173) in the CMVig group. By 14 days post-treatment, the median B cell count in both groups had dropped to undetectable levels. At the 12-month follow-up, repopulation remained limited, with median B cell counts of 10 cells/μL (IQR: 7–14) in the PET group and 8.5 cells/μL (IQR: 3–15) in the CMVig group, both of which remained below the normal lower limit (**Figure 5**). These findings demonstrate that rituximab-induced B cell depletion persists over an extended period in both groups.

### 3.5 Other secondary outcomes

The overall incidence of infection and rejection complications following kidney transplantation showed no significant differences between the two groups. T cell-mediated rejection (TCMR) occurred in 2.00% (1/50) of patients in the CMVig group and 2.12% (1/47) of patients in the PET group. No cases of antibody-mediated rejection (ABMR) were reported in either group. The incidence of pulmonary infections was 10.00% (5/50) in the CMVig group and 8.51% (4/47) in the PET group. BK virus-associated complications, including viruria and viremia, were comparable between the two groups. Additionally, no cases of herpes simplex virus (HSV-1, HSV-2) or varicella-zoster virus (VZV) infections were observed in either group (**Table 3**).

**Table 3:** Other secondary outcomes.

	preemptive therapy(n=47)	preemptive therapy+CMVig(n=50)
T cell-mediated rejection, TCMR (n)	1 (2.12%)	1 (2.00%)
Antibody-mediated rejection, ABMR (n)	0	0
Pulmonary infection (n)	4 (8.51%)	5 (10.00%)
BK virus viruria (n)	15 (31.9%)	18 (36.0%)
BK virus viremia (n)	5 (10.6%)	4 (8.0%)
BK virus-associated nephropathy (n)	0	1 (2%)
Varicella zoster virus infection (n)	0	0
Herpes simplex virus,HSV-1 HSV-2 (n)	0	0

## 4 Discussion

Cytomegalovirus (CMV), or human herpesvirus 5 (HHV-5), is a member of the Herpesviridae family that establishes lifelong latency following primary infection, with reactivation occurring under conditions of immunosuppression (16, 17). CMV infection represents a major challenge in post-transplant management, with infection rates reported as high as 30–67% in solid organ transplant recipients, depending on the transplanted organ and the intensity of the immunosuppressive regimen (5, 18, 19). In ABOi kidney transplantation, the need for enhanced immunosuppressive protocols and preconditioning regimens poses additional challenges in CMV management (20, 21). These desensitization protocols are designed to lower anti-A/B antibody (isoagglutinin) levels to a safe threshold to prevent rejection but inadvertently weaken immune defenses, significantly increasing susceptibility to opportunistic infections such as CMV (2, 22). These considerations underscore the critical need for tailored infection prevention strategies specifically for ABOi transplant recipients.

In some cases, CMVig has demonstrated efficacy and potential in preventing and treating CMV infections following ABOi kidney transplantation (23, 24).Mechanistic studies also suggest that CMVig could be a promising therapeutic candidate. In previous studies on CMVig, its multifaceted protective mechanisms have been documented. CMVig provides passive immunity by neutralizing circulating CMV particles (25). Additionally, CMVig enhances the body’s antiviral immune response while suppressing excessive immune activation, thereby balancing immune responses (5, 26). This dual modulation, involving both innate and adaptive immunity, facilitates viral clearance in high-risk populations, making it particularly crucial for ABO-incompatible kidney transplant recipients (25, 26). However, its specific clinical efficacy in ABOi kidney transplant recipients remains to be comprehensively observed and reported.

Our study demonstrates that prophylactic use of CMVig significantly reduces the incidence of CMV infection within 12 months. Patients receiving CMVig treatment showed a significantly lower incidence of clinically relevant CMV viremia compared to the preemptive therapy group. This finding is consistent with prior studies on solid organ transplantation. For instance, a systematic review and meta-analysis conducted by Barten et al. found that CMVig prophylaxis significantly reduced CMV infection rates in solid organ transplant recipients, reporting an infection rate of 35.8% in the CMVig group compared to 41.4% in the PET group (27).However, conflicting evidence exists. For instance, a randomized double-blind trial conducted by Ishida JH indicated that although CMVig delayed the onset of CMV viremia in some kidney transplant recipients, the difference compared to the placebo group was not statistically significant (28). The authors attributed these findings to factors such as insufficient sample size, selection bias in the study population, and inadequate follow-up duration.

Beyond reducing infection rates, CMVig delayed the onset of CMV viremia in our cohort, highlighting its potential for controlling CMV infection during the critical early post-transplant period. This extended protection may reduce reliance on antiviral drugs, thereby minimizing associated adverse effects such as nephrotoxicity and bone marrow suppression. Our clinical findings align with the mechanistic evidence supporting the therapeutic potential of CMVig, further reinforcing its role as a promising preventive strategy in ABOi kidney recipients.

CMV infection is a well-documented contributor to graft dysfunction and loss. Prior studies by Hellemans et al. and Ishikawa et al. have established a significant correlation between CMV infection and progressive declines in graft function (29, 30). Consistent with these findings, our one-year follow-up revealed that patients with clinically relevant CMV viremia exhibited significantly lower eGFR at multiple time points compared to those without, indicating sustained graft dysfunction attributable to CMV. These findings further underscore the critical importance of early CMV detection and intervention to prevent sustained graft damage and optimize transplantation outcomes.

Thus, we also investigated whether prophylactic CMVig directly enhances graft function. During the early follow-up period (1, 3, and 6 months), no significant differences in eGFR or serum creatinine levels were observed between the CMVig-treated and the preemptive group, suggesting limited direct protective effects of CMVig on graft function. However, by the 12-month follow-up, patients receiving CMVig demonstrated significantly improved eGFR and serum creatinine levels compared to patients in preemptive group. These improvements are likely attributable to the reduced incidence of CMV infection in the CMVig-treated group, which indirectly mitigated CMV-induced graft damage rather than reflecting a direct protective effect of CMVig itself.

Finally, our study evaluated the reconstitution of immune function and explored alternative desensitization strategies in ABOi kidney recipients. Previous studies, including those by Thiel and Colucci, have shown that peripheral B cell counts typically recover within 6 to 12 months following rituximab treatment (31, 32). However, persistently low B cell counts were observed throughout the entire observation period in our cohort. This prolonged immunosuppression likely accounts for the increased susceptibility to CMV infection observed in ABOi recipients (33). To address this, we explored a modified desensitization strategy in four patients, reducing rituximab doses from 200mg to 100 mg and administering a single preoperative dose of eculizumab. This C5 complement inhibitor supports desensitization by suppressing complement-mediated humoral immunity (34). While its efficacy is well-documented in atypical hemolytic uremic syndrome (aHUS), its role in preventing ABOi-associated antibody-mediated rejection (ABMR) remains uncertain (35, 36). Given that ABOi desensitization involves multiple immune pathways, including complement-dependent mechanisms and antibody-dependent cellular cytotoxicity (ADCC), relying solely on complement inhibitors may not be sufficient (37). Notably, none of the four patients experienced CMV infections or ABMR. These findings suggest that personalized desensitization protocols incorporating alternative strategies may help balance infection risk and immunosuppression in ABOi kidney transplantation.

Of course, this study has certain limitations. First, as a single-center study, the generalizability of the findings to other populations or different clinical settings may be limited. Second, the relatively short follow-up period restricts observations of long-term outcomes, such as graft survival and CMV-related mortality.

## 5 Conclusions

In conclusion, CMVig represents a promising prophylactic option for reducing CMV viremia incidence and delaying infection onset in ABOi kidney transplant recipients. Additionally, personalized desensitization protocols may further enhance CMV management and improve long-term outcomes for this high-risk population. However, further multi-center studies with extended follow-up periods are needed to validate these findings and establish optimized protocols for integrating CMVig into clinical practice.

## Data Availability

The original contributions presented in the study are included in the article/**Supplementary Material**. Further inquiries can be directed to the corresponding authors.

## Supplementary material

Supplementary Figure 1 Urinary proteins positive percentage over time. The bar chart shows the percentage of patients with urinary protein positivity at 1,3,6 and 12 months post-kidney transplantation (KT).

Supplementary Table 1 Donor-recipient relationships and surgical methods.
