# Supplementary material for: Impact of prophylactic cytomegalovirus immunoglobulin on cytomegalovirus viremia and graft function in ABO-incompatible living donor kidney transplantation: a retrospective analysis
Source: Front Immunol. 2025 Apr 28;16:1562951. doi: 10.3389/fimmu.2025.1562951 (PMC12066264; doi:10.3389/fimmu.2025.1562951)
Supplement: Supplementary Figure 1 — Urinary proteins positive percentage over time. The bar chart shows the percentage of patients with urinary protein positivity at 1,3,6 and 12 months post-kidney transplantation (KT). [file Table2.docx]

Supplementary Material

# Supplementary Figures


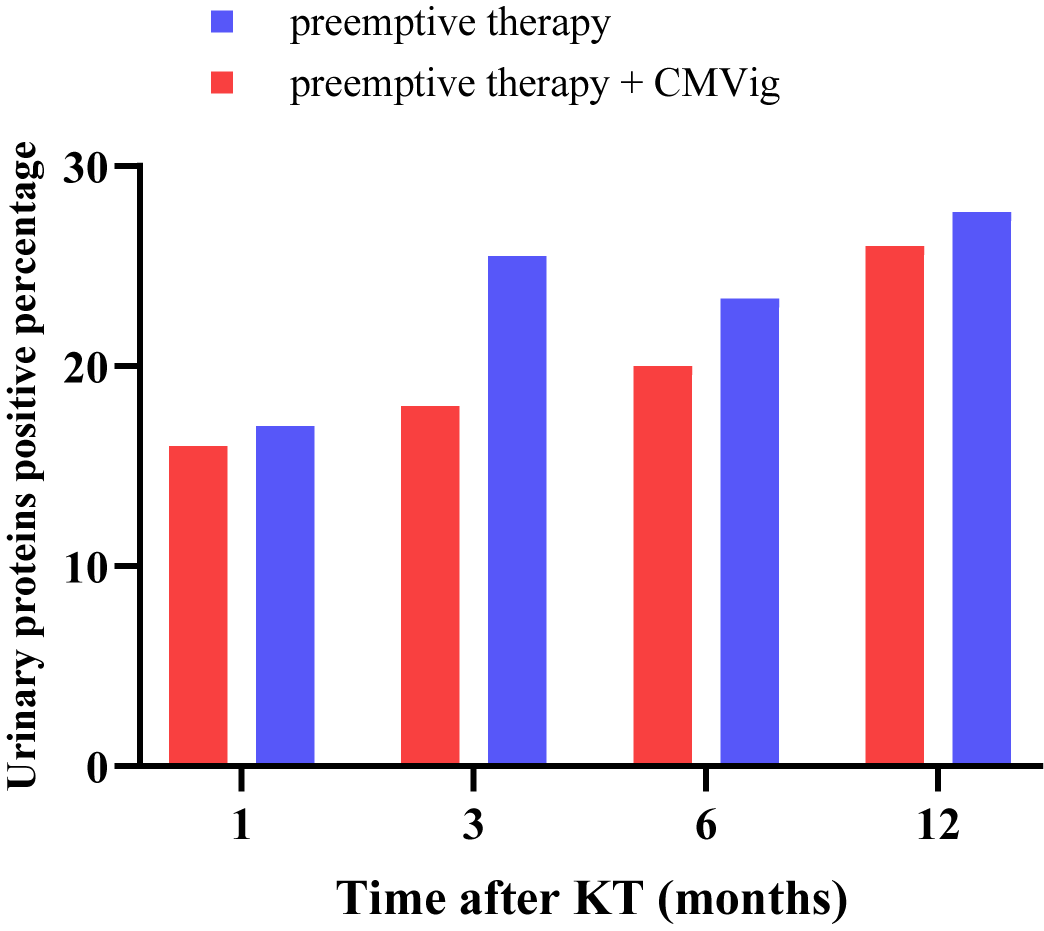


**Figure S1 Urinary proteins positive percentage over time.** The bar chart shows the percentage of patients with urinary protein positivity at 1,3,6 and 12 months post-kidney transplantation (KT).
