# Supplementary material for: Impact of prophylactic cytomegalovirus immunoglobulin on cytomegalovirus viremia and graft function in ABO-incompatible living donor kidney transplantation: a retrospective analysis
Source: Front Immunol. 2025 Apr 28;16:1562951. doi: 10.3389/fimmu.2025.1562951 (PMC12066264; doi:10.3389/fimmu.2025.1562951)
Supplement: Supplementary Table 1 — Donor-recipient relationships and surgical methods. [file Table1.docx]

Supplementary Table

|  | preemptive therapy（n=47） | preemptive therapy+CMVig（n=50） | p-value |
| --- | --- | --- | --- |
| Selection of ABOi-KT relative living donors |  |  | P=0.088 |
| genetically related (n,%) | 39(83.0) | 34(68.0) |  |
| non-genetically related (n,%) | 8(17.0) | 16(32.0) |  |
| Living donor nephrectomy |  |  | P=0.781 |
| Open donornephrectomy (n,%) | 25(53.2) | 28(56.0) |  |
| Retroperitoneoscopic laparoscopic  living donor nephrectomy (n,%) | 22(46.8) | 22(44.0) |  |
| CMVig = Cytomegalovirus hyperimmune globulin,ABOi-KT = ABO-incompatible kidney transplantation | | | |
